# Results of 30 reverse shoulder prostheses for revision of failed hemi- or total shoulder arthroplasty

**DOI:** 10.1007/s00590-013-1332-9

**Published:** 2013-10-18

**Authors:** Philippe Valenti, Alexandre Sahin Kilinc, Philippe Sauzières, Denis Katz

**Affiliations:** 1 Institut de la Main, Clinique Jouvenet, 6 Square Jouvenet, 75016 Paris, France; 2 Clinique du Ter, BP 71, 56275 Ploemeur, Morbihan, France

**Keywords:** Reverse shoulder prostheses, Shoulder pain, Revision, Massive cuff tear, Hemiarthroplasty, Total shoulder arthroplasty

## Abstract

**Purpose:**

Revision surgery for shoulder prosthesis remains a difficult task in shoulder surgery. The purpose of this retrospective study was to evaluate the clinical and radiological outcomes of a series of 30 reverse shoulder prostheses performed as revision of failed hemi- or total shoulder arthroplasty. The most relevant technical points in surgery are described, as are other surgical options; a rational strategy for the treatment of these patients is proposed.

**Materials and methods:**

Thirty patients (average age 69.5) were included. Mean follow-up was 36.4 months (range 24–100 months). There were 14 patients in group 1 (Delta III) and 16 in group 2 (Reverse Arrow).

**Results:**

A total of 83 % were satisfied (16 cases) or very satisfied (9 cases), and 17 % were disappointed (5 patients). The mean Constant score increased from a mean of 25–52. The mean score for pain improved from 5 (range 0–15) to 13 (range 5–15) (*p* < 0.001). The mean score of strength improved from 1 (range 0–6) to 5 (range 0–10) (*p* < 0.001). The forward elevation changed from a mean of 55° (range 0–120) to 108° (range 40–160) (*p* < 0.001). There was no significant improvement of external rotation at 0° abduction (range 14°–18°) or internal rotation (range 5–4.63). There were 4 scapular notching. We could not find the influence of scapular notching on Constant Score. Complication rate was 26.6 %.

**Conclusion:**

Reverse total shoulder arthroplasty prosthesis represents an available option in difficult cases of failed hemiarthroplasty or total shoulder arthroplasty when the rotator cuff is irreparable and the glenoid bone stock is sufficient.

**Level of evidence:**

Level 4.

## Introduction

Revision surgery for shoulder prostheses remains among the most difficult tasks in shoulder surgery. The poor results in function and longevity of unconstrained prosthesis for revision of failed hemiarthroplasty or total shoulder arthroplasty led many authors to use reverse ball and socket prosthesis for selected cases [1]. Many factors (quality of the subscapularis muscle, function of the cuff, quantity and the quality of the glenoid bone, deltoid muscle condition) can explain the poor results of revision with an anatomic arthroplasty [2–6].

In 1980, the biomechanical works of Paul Grammont proved that medialization and lowering of the center of rotation can increase the level arm of the deltoid and restore active forward elevation for pseudoparalytic shoulder [7]. Reverse shoulder prosthesis (RSP) has been used by many authors in irreparable cuff tear and also in other indications [6, 8–11]. Since the indication for this prosthesis are widening, we wanted to determine whether the RSP is effective in shoulder prosthetic revision surgery.

The purpose of this retrospective study was to examine the clinical and radiological outcomes of a series of 30 reverse shoulder prostheses performed as revision of failed hemi- or total shoulder arthroplasty. Secondary aim was to comparing the results of a less medialized reversed Arrow shoulder prosthesis with those of the traditional reverse Delta III prosthesis in terms of active external rotation and development of glenoid notch.

The most relevant technical points in surgery are described, as are other surgical options; a rational strategy for the treatment of these patients is proposed.

## Materials and methods

### Patient selection

Between 1998 and 2005, 35 consecutive patients underwent revision of failed hemiarthroplasty or total shoulder arthroplasty at our institutions. All the procedures were performed by three senior surgeons. Four patients were lost to follow-up, and one patient died. The study included 30 patients (18 women and 12 men) with a preoperative mean follow-up of 36.4 months (range 24–100 months). The right side was involved 17 times and the left side 13 times. The dominant side was affected in 65 % of cases. At the time of revision surgery, the mean age of the patients was 69.5 years (range 50–85 years). Twenty-five shoulders were converted from a failed hemiarthroplasty, 4 shoulders were revised for a failed total shoulder arthroplasty, and one shoulder was converted from a failed bipolar shoulder arthroplasty. The mean delay between the first arthroplasty and the revision was 2.6 years (range 1–6 years). The mean postoperative follow-up was 63.4 months (range 49–100 months).

In the failed hemiarthroplasty group (25 cases), the original surgical indication was acute 4 part fracture of the proximal humerus (11 cases), massive cuff tear in old patients with painful forward elevation greater than 120° (7 cases), malunion of the proximal humerus (3 cases), gleno-humeral arthritis with a reparable cuff tear (3 cases) and necrosis (1 case). The reasons for the revision surgery in this group were stiff and painful shoulder (9 cases), migration of the tuberosities (5 cases), secondary rotator cuff tear (4 cases), malposition of the component (3 cases), erosion of the glenoid (2 cases), humeral loosening (1 case) and complication of infection (1 case).

In the failed total shoulder arthroplasty, the original surgical indications were gleno-humeral arthritis with a functional rotator cuff (2 cases, both treated with unconstrained arthroplasty) and 2 cases of rotator cuff deficiency (both treated with reverse shoulder prosthesis). The reasons of the revision surgery in this group were glenoid loosening associated with a cuff tear with an upper migration of the humeral and infection after reverse prosthesis.

The original bipolar shoulder prosthesis was implanted following an acute fracture dislocation of the humeral head. The revision surgery for this patient was indicated for chronic dislocation of the prosthesis with lack of active elevation.

Before shoulder arthroplasty, 24 had only one previous surgery (group A) and 6 patients had more than 2 surgeries (group B).

Inclusion criteria for revision with a reverse shoulder arthroplasty were an irreparable cuff tear associated with a good glenoid bone stock (sufficient to implant a metal back and a semi-constrained prosthesis). Exclusion criteria were the following: deltoid palsy and active infection. Two types of prosthesis were used for the revision: before 2003 the Delta III (DePuy orthopaedics, Warsaw, IN) in 14 cases and after 2003 the reverse Arrow (FH orthopedics, Mulhouse, France) in 16 cases.

### Surgical technique

All the patients were operated under general anesthesia with an interscalene block in beach-chair position. We used an anterosuperior transdeltoid approach in 14 cases and a deltopectoral approach in 16 cases. The anterosuperior approach was used during the primary hemiarthroplasty for acute fracture as well as for the revision surgery between 1998 and 2003. With this approach, 3 diaphyseal fractures of the humerus occurred during extraction of the humeral stem or cement removal. After 2003, we preferred a deltopectoral approach in all cases independently from the first approach. The long head of the biceps was previously cut or fixed onto the lesser tuberosity. The rotator cuff was continuous in 7 cases, but it was thin and fibrotic and considered as nonfunctional. Through the deltopectoral approach, 6 humeral osteotomies were necessary to facilitate the extraction of the humeral stem and three distal windows to remove distal plug of cement. Cerclage wire was required to stabilize the diaphyseal osteotomy. If the cortical bone of the diaphysis was too thin, we preferred a cemented small humeral stem (size 8) into the mantle of cement to prevent any fracture or perforation of the diaphysis with the risk for the cement to run away.

With the Delta III prosthesis, the design and length (100 mm standard) of the humeral stem persuaded us to always fix it with cement. Metaphyseal cortico-cancellous bone graft was preferred to modular spacer neck in situation of sequelae of fracture with proximal bone loss. In 13 cases, we used a standard polyethylene cup, whereas in 3 cases, we used polyethylene modular spacer neck to stabilize the prosthesis (2 cases with +5 mm and one with +10 mm). We decided not to use a retentive cup, which is more constrained. Instead in five cases, we preferred a 39-mm glenosphere to lateralize the humerus for retensioning the deltoid. When, in the group of fracture sequelae, the reason for failure of hemiarthroplasty was malpositioning of the humeral component, the difficulty was to reproduce “ideal length” for good tension of the deltoid and preoperative contralateral scale was mandatory.

With the reverse Arrow prosthesis, a press fit metaphyseal stem with cancellous and chips bone graft allowed to avoid cement in 6 cases. In all 14 cases, we used a standard polyethylene cup. No 5 or 10 modular neck was necessary to stabilize the prosthesis.

In 4 cases associated with a small size component, a central defect in the glenoid surface was grafted with cancellous bone graft from iliac crest. The integrity of the posterior and anterior allowed a primary press fit fixation. In two cases, the anterior wall was repaired by means of two cortico-cancellous bone graft fixed with two screws. The design of metal back of reverse Arrow prosthesis with a central keel and an anterior lug improves the primary press fit fixation. A complementary anteroposterior screw, which crosses the anterior lug and keel to end at the posterior wall, was used in 3 cases. This locks the base plate, stabilizes bone graft and increases the potential of healing bone.

### Postoperative rehabilitation

All patients were managed with a simple sling postoperatively, with the arm at the side during 4 weeks to allow healing of anterior deltoid, and passive range-of-motion exercises were started the day after surgery. Active-assisted activities and active range of motion were initiated after 4 weeks.

### Patient assessment

Clinical evaluation was performed before operation and after surgery using the Constant–Murley Assessment. Ranges of active and passive movements estimated visually were recorded for forward elevation and abduction, external rotation with the arm at the side (ER1), external rotation in 90° of abduction (ER2) and for internal rotation.

Postoperative radiological evaluation included a true anteroposterior view of the gleno-humeral joint in neutral rotation under fluoroscopic control. We looked for evidence of glenoid component loosening (radiolucent lines around the base plate, hardware breakage and change in base plate position) or scapular notching (graded according to the Nerot classification).

### Statistical analysis

Statistical analysis was performed on preoperative and postoperative data by use of descriptive statistics, as well as the Student’s *t* test or Mann–Whitney test for continuous data. Level of confidence was 95 %.

## Results

### Clinical and radiological results

The mean Constant score increased from a 25 (range 21–28) to 52 (range 48–55) (*p* < 0.001). The mean score for pain improved from 5 (range 0–15) to 13 (range 5–15) (*p* < 0.001). The mean forward elevation changed from 55° (range 0–120) to 108° (range 40–160) (*p* < 0.001). There was no significant improvement of external rotation at 0° abduction (14°–18°) and internal rotation (5–4.63). The mean score of strength improved from 1 kg (range 0–6) to 5 kg (range 0–10) (*p* < 0.001) (Table 1).

**Table 1:** Clinical results for pain, activity, range of motion and strength

	Pre-op	Post-op	*p* value
Constant	24.47 (8–46)	51.57 (30–67)	<0.001
Pain	4.77 (0–15)	13.1 (5–15)	<0.001
Activity	5.97 (2–14)	11.97 (6–19)	<0.001
Strength	0.83 (0–6)	5.23 (0–10)	<0.001
Flexion (°)	55.2 (0–120)	107.5 (40–160)	<0.001
ER1 (°)	14.3 (−10 to 60)	18.17 (−10 to 60)	0.2912
IR (CST)	5 (2–10)	4.63 (0–8)	0.61

For the subjective results, 83 % were satisfied (16 cases) or very satisfied (9 cases) and 17 % were disappointed (5 patients). Among the five patients who had been disappointed, three had complications (one inferior dislocation, one humeral loosening with a deep infection reoperated and one deltoid palsy).

No statistically significant difference was observed in terms of Constant score between the patients in group A (one previous surgery) and group B (more than two previous surgeries) (Table 2).

**Table 2:** Results according to the number of previous operation

Number of previous surgeries	Constant score Mean value [range in parentheses; 95 % confidence interval (CI)]			
	Pre-op	Post-op	Gain	*p* value
Group A: 1 previous intervention (*n* = 24)	25 (21–28)	51 (47–55)	26 (22–31)	<0.001
Group B: ≥2 previous interventions (*n* = 6)	24 (13–35)	54 (45–63)	30 (25–35)	<0.001
Series (*n* = 30)	25 (21–28)	52 (48–55)	27 (22–32)	<0.001

According to the etiologies of primary prosthesis, there was no statistically significant difference in terms of Constant score (Table 3).

**Table 3:** Results according to the initial indication for surgery: constant score

	Constant score Mean value (range in parentheses; 95 % CI)			
	Pre-op	Post-op	Gain	*p* value
Cuff tear (*n* = 14)	30 (23–36)	54 (47–60)	24 (20–28)	<0.001
Fracture, fracture sequelae, dislocation or necrosis (*n* = 16)	23 (18–27)	51 (46–55)	28 (22–34)	<0.001
Series (*n* = 30)	25 (21–28)	52 (48–55)	27 (22–32)	<0.001

The patients with sequelae of fracture had better increase in forward active flexion than the patients with massive cuff tear, who, vice versa, had better absolute postoperative Constant score (Table 4).

**Table 4:** Results according to the initial surgery: forward active elevation

	Forward active flexion (°) Mean value (range in parentheses; 95 % CI)			
	Pre-op	Post-op	Gain	*p* value
Massive cuff tear (*n* = 14)	69 (45–93)	111 (98–123)	42 (27–57)	<0.05
Fracture, fracture sequelae, dislocation or necrosis (*n* = 16)	47 (30–63)	102 (85–118)	55 (35–75)	<0.001
Series (*n* = 30)	55 (44–67)	108 (99–116)	53 (41–65)	<0.001

**Fig. 1:**
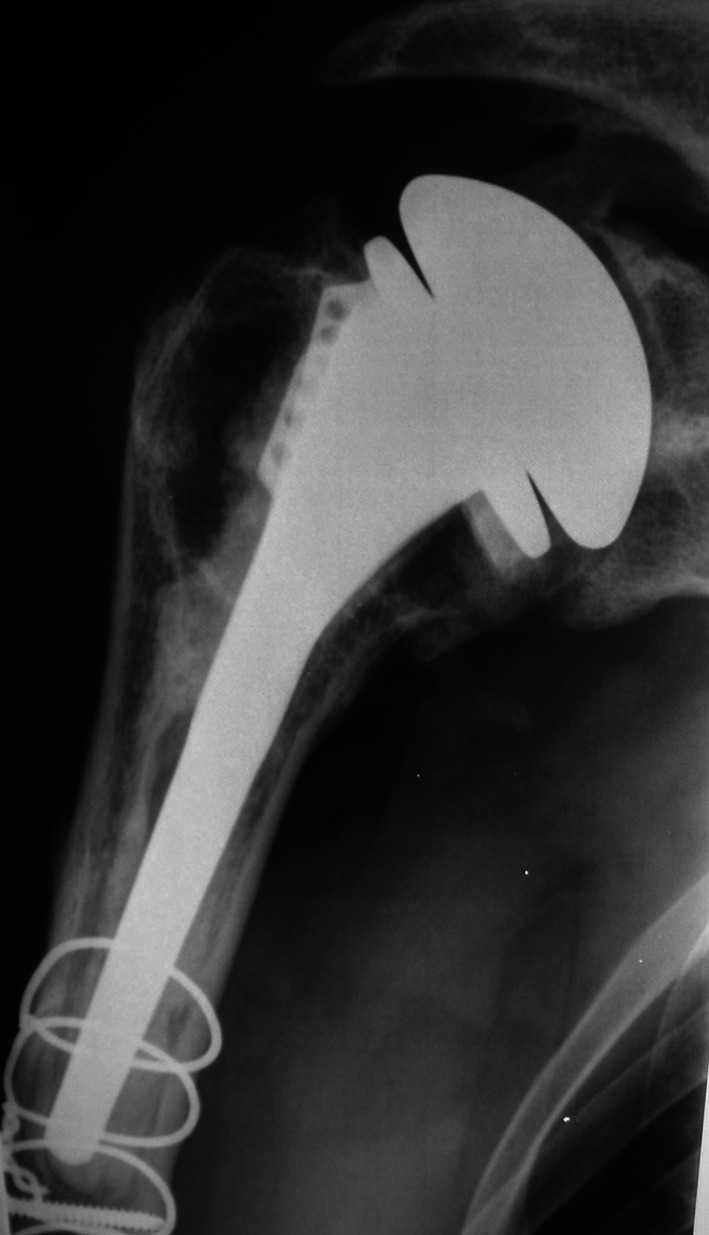
Male patient, 55 years old, operated 7 years ago for a centered osteoarthritis of the right shoulder. A fracture of the humeral stem occurred during the first procedure and was treated with cerclages. After 7 years, the patient was painful, and he developed a glenoidite with an exenteration of the head

**Fig. 2:**
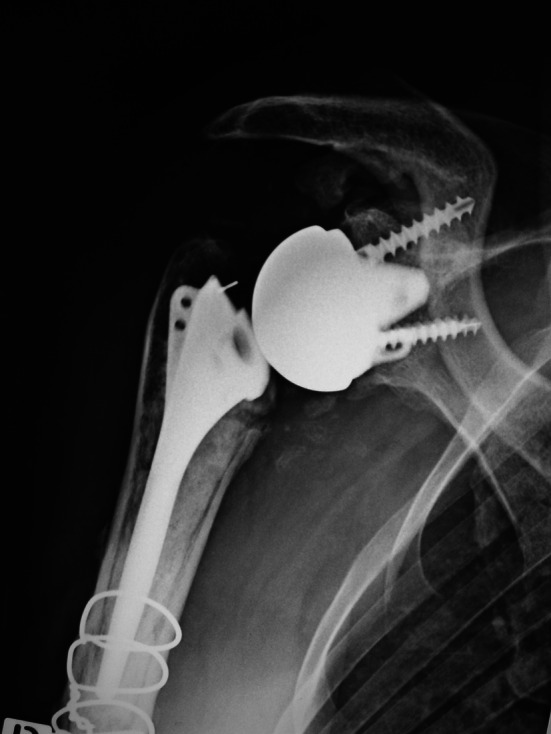
Same patient, revision was made with a cemented *Reverse Arrow total arthroplasty*

**Fig. 3:**
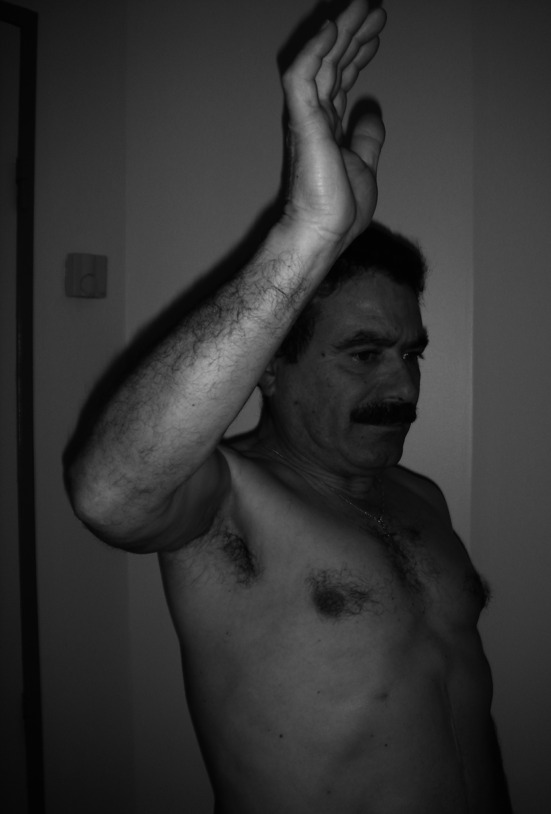
Same patient, 4-year follow-up, constant score = 65 (15, 12, 24, 14)

Among the revision surgeries, there was no significant difference in terms of functional outcome between the deltopectoral approach and the superolateral approach. The mean postoperative Constant score was 50 (range 45–55) in deltopectoral approach and 53 (range 49–57) in superolateral approach (Figs. 1, 2, 3).

No statistically significant difference was observed in terms of Constant score and forward active flexion between the two types of reverse prosthesis, but the gain in external rotation at 0° of abduction was significantly more important in the group with reverse Arrow prosthesis (+9° *p* < 0.05) (Tables 5, 6, 7).

**Table 5:** Clinical results according to the type of reverse prosthesis in terms of constant score

	Constant score Mean value (range in parentheses; 95 % CI)			
	Pre-op	Post-op	Gain	*p* value
Group Delta III (*n* = 14)	25 (19–30)	51 (45–56)	27 (21–33)	<0.001
Group Arrow (*n* = 16)	24 (19–29)	52 (48–56)	28 (23–32)	<0.001
Series (*n* = 30)	25 (21–28)	52 (48–55)	27 (22–32)	<0.001

**Table 6:** Clinical results according to the type of reverse prosthesis in terms of active forward elevation

	Forward active flexion (°) Mean value (range in parentheses; 95 % CI)			
	Pre-op	Post-op	Gain	*p* value
Group Delta III (*n* = 14)	61 (42–79)	112 (100–124)	56 (40-72)	<0.001
Group Arrow (*n* = 16)	50 (35–66)	103 (89–117)	53 (36–68)	<0.001
Series (*n* = 30)	55 (44–67)	108 (99–116)	53 (41–65)	<0.001

**Table 7:** Clinical results according to the type of reverse prosthesis in terms of external rotation

	ER1 (°) Mean value (range in parentheses; 95 % CI)			
	Pre-op	Post-op	Gain	*p* value
Group Delta III (*n* = 14)	21 (11–32)	20 (11–29)	−1 (−12 to 10)	0.8
Group Arrow (*n* = 16)	7 (2–12)	16 (9–24)	9 (1–17)	0.05
Series (*n* = 30)	14 (8–20)	18 (13–24)	4 (−3 to 11)	0.29

The postoperative radiographic findings showed evidence of scapular notching (4 cases) less than one-year follow-up in the group of Delta III (28 %): these were classified, according to Nérot [12], as grade 2 (one case), grade 3 (2 cases) and grade 4 (one case). In this small series, we found no significant correlation between scapular notching and Constant Score. No scapular notching was identified in the group of Arrow. The assessment of the axis of the new humeral stem showed 30 % of varus deformity: all the cement was not removed, and a thin stem was introduced into the mantle in 10 cases. At last follow-up, we did not revise any Delta prosthesis for glenoid loosening.

### Complications

Complications occurred in 8 shoulders (26.6 %) both intraoperatively and postoperatively, but only 5 shoulders required reoperations (16.6 %), with final results similar to the noncomplicated cases.

The three intraoperative complications were humeral diaphysis fractures during extraction of the cement through a superolateral approach.

The five postoperative complications were two material disassembly, two dislocations and one postoperative infection.

## Discussion

Causes of revision for hemiarthroplasty and total shoulder arthroplasty have been largely reported in the literature [13–18]. In the group of hemiarthroplasty (25 cases in our study), tuberosities problems (migration, malunion and nonunion) are the main causes of failure [19–22]. In descending order of frequency, we can encounter glenoid erosion, secondary rotator cuff tear, instability and infection. Sometimes, these complications can be associated with rotator cuff deficiency and instability. In the group of total shoulder arthroplasty (only 5 cases in our study), the main causes of failure were glenoid loosening, rotator cuff deficiency and infection. In 1994, Wirth and Rockwood reported the causes of failure of total arthroplasty in order of frequency based on a meta-analysis of 1,615 cases: glenoid loosening, instability, rotator cuff tear, periprosthetic fracture, infection, implant failure and deltoid dysfunction [4].

Not many surgical options are available for failed shoulder prosthesis, especially when worsened by a loss of glenoid bone stock or an irreparable rotator cuff lesion.

Before using reverse shoulder prosthesis, many authors reported the difficulties and worse results obtained with an unconstrained prosthesis [23].

Petersen et al. [2] used an anatomic Neer-type prosthesis in their series of 158 cases operated in a 15-year period. They reported 19 % of excellent results, 41 % of satisfactory results, 28 % of unsatisfactory and 12 % of poor results. The high rate of bad results (40 %) explains why this treatment is not the preferred one.

In the experience of Lee et al. [24] with the bipolar prosthesis in 14 patients, the poor elevation achieved with the procedure limits all enthusiasm.

Arhens et al. reported 60 % of recurrence of anterior instability and 40 % of recurrence of posterior instability in revision of instability after unconstrained arthroplasty [25, 26]. Sanchez-Sotelo reported only 40 % of success on a series of 33 hemiarthroplasty [15].

For the cases with severe bone loss, a gleno-humeral fusion might be proposed, with the well-known limitations posed by this kind of treatment. In cases of intractable pain, shoulder arthrodesis might be regarded as the sole choice to alleviate the patient with some functional expectation [27]. Resection arthroplasty has been also proposed for the cases of virulent infections and extensive bone loss [28]. However, the procedure renders the shoulder useless in practical terms. For those patients with a hemiarthroplasty or a total arthroplasty in whom a good rotator cuff is found, a new glenoid component is the procedure of choice, provided the glenoid resurfacing with autologous bone graft in cases where subchondral bone defects are found [1, 16, 23]. We concluded that reverse shoulder prosthesis with the Delta III and reverse Arrow is a good option for revision of failed shoulder prosthesis. The best indications are irreparable cuff tear with subsequent upward migration of the humeral head, absence of the coracoacromial arch and anterior instability with extensive lesion of the cuff. The results of reverse shoulder prosthesis for revision of hemiarthroplasty or total shoulder arthroplasty are better than those obtained with an unconstrained prosthesis in terms of active forward elevation and stability of the prosthesis. Overall patients, in the current series, had 27 points of improvement in terms of the Constant score and 53° of improvement in terms of active elevation, which are gains that are comparable with previous reports [8, 10, 29]. The gains of the reverse shoulder prosthesis are similar to those obtained with the other etiologies as cuff tear arthropathy or osteoarthritis with massive cuff tear or massive cuff tear alone, but the final level of performance is low in terms of Constant score and forward active elevation. The gain in external rotation at 0° of abduction was limited in our series (4°) and overall not significant but was considerably more important in the group with Arrow prosthesis (+9° *p* < 0.05), in which the center of rotation is more lateralized than the Delta III.

Using a reverse prosthesis with a center of rotation lateral to the level of the glenoid (Encore, Houston, Texas), Frankle et al. [9] reported encouraging results, in a series of sixty patients. The gain was 35.9° in external rotation at 0° of abduction compared with the 11.2° in the study reported by Sirveaux et al. [30] with a Delta III. The first hypothesis is that a more anatomic center of rotation improves the tension in the remaining subscapularis or infraspinatus or teres minor muscles, recruiting some anterior and posterior fibers of the deltoid, which participate to external and internal rotation.

The second hypothesis is that the lateral position of the humerus decreases the risk of impingement with the inferior and posterior part of the scapula. Glenoid notching was absent in the group of 16 reverse Arrow and accounted for 28 % in the group of Delta III prosthesis. Frankle et al. in 2005 reported no medial encroachment or progressive erosion of the glenoid; seven patients (12 %) required revision for glenoid loosening at a mean postoperative follow-up of 21.4 months. The Author found no osseous in-growth into the baseplate, and the failure appeared to be due to metal fatigue of the screws. Similarly, Boileau proposed a bony increased offset reverse shoulder arthroplasty (BIO-RSA) as a biologic solution to avoid scapular notching prosthetic instability and to improve shoulder rotation [31].

The complication rate in the current study (26.6 %, 8/30) was lower than in other series (range 13.3–52 %) [8–10, 30, 32]. As for the technical details, the deltopectoral approach facilitates the extraction of both humeral stem and cement, thus preventing the fracture of the diaphysis. A thin stem can be cemented directly into the mantle without the risk of fracture of the humerus during extraction of the cement. Autogenic bone graft from the iliac crest was our choice in metaphyseal bone loss although a humeral spacer can be an excellent alternative. In revision surgery of hemiarthroplasty for complex fracture, the metaphyseal allograft for the reconstruction of the proximal part of the humerus always disappeared before 2-year follow-up. The goal during this revision is to improve the strength of the deltoid and his power of coaptation of the joint to avoid dislocation of the shoulder and to recover active anterior elevation and external rotation for the quality of daily activity. A less medialized reverse prosthesis is more adapted in revision cases to reduce the rate of complication and particularly the potential instability. However, the concave shape of the baseplate should ensure a primary press fit fixation to resist the shearing forces. A central glenoid defect can be overcome by cancellous bone grafting, and the cortico-cancellous bone grafting is useful to bring a primary press fit of the baseplate during the reconstruction of anterior or posterior wall of the glenoid. The procedure is contraindicated in cases of extensive loss of the glenoid bone, chronic and/or highly virulent infections, and deltoid palsy.

The present study had several limitations. The size of the two groups of prosthesis is too small to advocate significant functional differences. The complication rate is also increased by the use of first-generation Reverse Arrow between 2003 and 2005 with mechanical failure between glenosphere and metal baseplate.

## Conclusions

The present study shows that the reverse total shoulder arthroplasty prosthesis represents an available option in difficult cases of failure of hemiarthroplasty or total shoulder arthroplasty when the rotator cuff is irreparable and the glenoid bone stock is sufficient. Patients are satisfied in terms of pain relief, gain in active elevation is significant, but there is no or moderate gain in ER1.

The success of this salvage procedure depends on an appropriate exposure, quality of the fixation of the glenoid component and the reproduction of the optimal tension of the deltoid muscle.

## References

[1] Gagey O, Pourjamasb B, Court C (2001). Revision arthroplasty of the shoulder for painful glenoid loosening: a series of 14 cases with acromial prostheses reviewed at 4 year follow up. Rev Chir Orthop Reparatrice Appar Mot.

[2] Petersen SA, Hawkins RJ (1998). Revision of failed total shoulder arthroplasty. Orthop Clin North Am.

[3] Rodosky MW, Bigliani LU (1996). Indications for glenoid resurfacing in shoulder arthroplasty. J Should Elbow Surg.

[4] Wirth MA, Rockwood CA (1994). Complications of shoulder arthroplasty. Clin Orthop Relat Res.

[5] Deshmukh AV (2005). Total shoulder arthroplasty: long-term survivorship, functional outcome, and quality of life. J Should Elbow Surg.

[6] Boileau P et al (2013) Revision surgery of reverse shoulder arthroplasty. J Shoulder Elbow Surg 22(10):1359–1370. doi:10.1016/j.jse.2013.02.004 10.1016/j.jse.2013.02.004 23706884

[7] Grammont PM, Trouilloud P, Laffay JP, Deries X (1987). Etude et réalisation d’une nouvelle prothèse d’épaule. Rhumatologie.

[8] Boileau P (2006). Neer award 2005: the Grammont reverse shoulder prosthesis: results in cuff tear arthritis, fracture sequelae, and revision arthroplasty. J Should Elbow Surg.

[9] Frankle M (2005). The reverse shoulder prosthesis for glenohumeral arthritis associated with severe rotator cuff deficiency. A minimum two-year follow-up study of sixty patients. J Bone Joint Surg Am.

[10] Wall B (2007). Reverse total shoulder arthroplasty: a review of results according to etiology. J Bone Joint Surg Am.

[11] Werner CM (2005). Treatment of painful pseudoparesis due to irreparable rotator cuff dysfunction with the Delta III reverse-ball-and-socket total shoulder prosthesis. J Bone Joint Surg Am.

[12] Valenti PH, Bouttens D, Nerot C (2001) Delta 3 reversed prothesis for osteoarthritis with massive rotator cuff tear, long term results (>5 years). Shoulder prosthesis…two to 10 years follow-up. Sauramps Medical, Montpellier, pp 253–259

[13] Cofield RH, Frankle MA, Zuckerman JD (1995). Humeral head replacement for glenohumeral arthritis. Semin Arthroplasty.

[14] Boyd AD, Aliabadi P, Thornhill TS (1991). Postoperative proximal migration in total shoulder arthroplasty. Incidence and significance. J Arthroplasty.

[15] Sanchez-Sotelo J (2003). Instability after shoulder arthroplasty: results of surgical treatment. J Bone Joint Surg Am.

[16] Hill JM, Norris TR (2001). Long-term results of total shoulder arthroplasty following bone-grafting of the glenoid. J Bone Joint Surg Am.

[17] Sperling JW, Cofield RH (1998). Revision total shoulder arthroplasty for the treatment of glenoid arthrosis. J Bone Joint Surg Am.

[18] Cooper RA, Brems JJ (1991). Recurrent disassembly of a modular humeral prosthesis. A case report. J Arthroplasty.

[19] Boileau P (2002). Tuberosity malposition and migration: reasons for poor outcomes after hemiarthroplasty for displaced fractures of the proximal humerus. J Should Elbow Surg.

[20] Bigliani LU (2002). Case challenges in shoulder surgery: what would you do?. J Arthroplasty.

[21] DiGiovanni J (1998). Hemiarthroplasty for glenohumeral arthritis with massive rotator cuff tears. Orthop Clin N Am.

[22] Gronhagen CM (2007). Medium-term results after primary hemiarthroplasty for comminute proximal humerus fractures: a study of 46 patients followed up for an average of 4.4 years. J Should Elbow Surg.

[23] Antuna SA (2001). Glenoid revision surgery after total shoulder arthroplasty. J Should Elbow Surg.

[24] Lee DH, Niemann KM (1994). Bipolar shoulder arthroplasty. Clin Orthop Relat Res.

[25] Ahrens P, Boileau P, Walch G (2001) Anterior instability after unconstrained shoulder arthroplasty. In: 2000 shoulder prothesis… 2–10 year follow up. Sauramps Médical

[26] Ahrens P, Boileau P, Walch G (2001) Posterior instability after unconstrained shoulder arthroplasty. In: 2000 shoulder prothesis… 2–10 year follow up. Sauramps Médical

[27] Cofield RH, Briggs BT (1979). Glenohumeral arthrodesis. Operative and long-term functional results. J Bone Joint Surg Am.

[28] Braman JP (2006). The outcome of resection shoulder arthroplasty for recalcitrant shoulder infections. J Should Elbow Surg.

[29] De Wilde LF, Audenaert EA, Berghs BM (2004). Shoulder prostheses treating cuff tear arthropathy: a comparative biomechanical study. J Orthop Res.

[30] Sirveaux F (2004). Grammont inverted total shoulder arthroplasty in the treatment of glenohumeral osteoarthritis with massive rupture of the cuff. Results of a multicentre study of 80 shoulders. J Bone Joint Surg Br.

[31] Boileau P (2011). Reply to letter to the editor: bony increased-offset reversed shoulder arthroplasty: minimizing scapular impingement while maximizing glenoid fixation. Clin Orthop Relat Res.

[32] Ortmaier R (2013). Reverse shoulder arthroplasty in revision of failed shoulder arthroplasty-outcome and follow-up. Int Orthop.

